# Palbociclib‐letrozole as first‐line treatment for advanced breast cancer: Updated results from a Japanese phase 2 study

**DOI:** 10.1002/cam4.3091

**Published:** 2020-05-18

**Authors:** Masato Takahashi, Norikazu Masuda, Reiki Nishimura, Kenichi Inoue, Shinji Ohno, Hiroji Iwata, Satoshi Hashigaki, Yasuaki Muramatsu, Yoshiko Umeyama, Masakazu Toi

**Affiliations:** ^1^ National Hospital Organization Hokkaido Cancer Center Hokkaido Japan; ^2^ National Hospital Organization Osaka National Hospital Osaka Japan; ^3^ Kumamoto Shinto General Hospital Kumamoto Japan; ^4^ Saitama Cancer Center Saitama Japan; ^5^ The Cancer Institute Hospital of JFCR Tokyo Japan; ^6^ Aichi Cancer Center Hospital Aichi Japan; ^7^ Pfizer R&D Japan Tokyo Japan; ^8^ Pfizer Japan Inc Tokyo Japan; ^9^ Kyoto University Graduate School of Medicine Kyoto Japan

**Keywords:** advanced breast cancer, cyclin‐dependent kinase, Japanese, letrozole, palbociclib

## Abstract

Palbociclib is a highly selective, reversible, oral inhibitor of cyclin‐dependent kinases 4 and 6 that is approved to treat hormone receptor‐positive/human epidermal growth factor receptor 2‐negative advanced breast cancer. An open‐label, single‐arm, Japanese phase 2 study was conducted to investigate the efficacy and safety of palbociclib plus letrozole as first‐line treatment in 42 postmenopausal patients with estrogen receptor‐positive/human epidermal growth factor receptor 2‐negative advanced breast cancer. The probability of progression‐free survival originally reported at 1 year was 75.0% (90% confidence interval, 61.3‐84.4), but median progression‐free survival was not attained at the primary analysis. In this report, updated efficacy and safety results with a longer follow‐up period are presented. The median duration of treatment in the updated analysis was 33.0 months (range, 1.8‐49.2). The probability of progression‐free survival at 1 year was 75.6% (90% confidence interval, 62.4‐84.7). Median progression‐free survival was 35.7 months (95% confidence interval, 21.7‐46.7). Objective response rate and disease control rate were 47.6% (95% confidence interval, 32.0‐63.6) and 85.7% (95% confidence interval, 71.5‐94.6), respectively. Common treatment‐related adverse events (all grades; grade 3/4) were neutropenia (100%; 93%), leukopenia (83%; 60%), and stomatitis (76%; 0%). Treatment‐related febrile neutropenia was reported in one patient. In general, no clinically meaningful deterioration in health‐related quality of life was observed. Palbociclib plus letrozole remained effective and tolerable in Japanese postmenopausal patients with estrogen receptor‐positive, human epidermal growth factor receptor 2‐negative advanced breast cancer in this updated analysis.

## Graphical abstract

With longer follow‐up of an open‐label, single‐arm, Japanese phase 2 study conducted to investigate the efficacy and safety of palbociclib plus letrozole as first‐line treatment in postmenopausal patients with estrogen receptor‐positive/human epidermal growth factor receptor 2‐negative advanced breast cancer, median progression‐free survival was 35.7 months (95% CI, 21.7‐46.7). Palbociclib plus letrozole remained effective and tolerable in Japanese postmenopausal patients with estrogen receptor‐positive/human epidermal growth factor receptor 2‐negative advanced breast cancer.

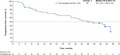

## 1 INTRODUCTION

The incidence of breast cancer has increased dramatically in Japanese women.^1^ It is now the most common cancer and one of the top 5 causes of cancer‐related deaths among Japanese women.^1^ Breast cancer survival rates are high when detected early, but 5‐year survival rates are much lower for Japanese women with advanced breast cancer (ABC).^2^ Cyclin‐dependent kinase 4/6 (CDK4/6) inhibitor treatment in combination with an aromatase inhibitor (AI) has become the standard of care as first‐line treatment in postmenopausal women with hormone receptor‐positive (HR+)/human epidermal growth factor receptor 2‐negative (HER2–) ABC.^3^ In September 2017, palbociclib, a highly selective, reversible, oral CDK4/6 inhibitor,^4^, ^5^ was approved in Japan for the treatment of unresectable or recurrent breast cancer.^6^

The results of an international, open‐label, phase 2 clinical trial (PALOMA‐1; NCT00721409) indicated that palbociclib plus letrozole prolonged progression‐free survival (PFS) vs letrozole alone (20.2 vs 10.2 months, respectively; hazard ratio, 0.49; 95% CI, 0.32‐0.75; one‐sided *P* = .0004) in postmenopausal women with estrogen receptor‐positive (ER+)/HER2– ABC; Japanese patients were not included in this study.^7^ However, Japanese patients were included in a phase 3, randomized, placebo‐controlled clinical trial of palbociclib plus letrozole as first‐line treatment in postmenopausal women with ER+/HER2– ABC (PALOMA‐2; NCT01740427).^8^, ^9^ In the overall study population, median PFS was significantly longer with palbociclib plus letrozole vs placebo plus letrozole (27.6 vs 14.5 months; hazard ratio, 0.56; 95% CI, 0.46‐0.69; one‐sided *P* < .0001; data cutoff date, 31 May 2017).^10^ Japanese patients were also included in a phase 3, randomized, placebo‐controlled trial of palbociclib plus fulvestrant vs placebo plus fulvestrant as treatment for patients with HR+/HER2– ABC who had disease progression on prior endocrine therapy (PALOMA‐3; NCT01942135).^11^, ^12^ In the overall study population, median PFS was also significantly longer with palbociclib plus fulvestrant vs placebo plus fulvestrant (11.2 vs 4.6 months, respectively; data cutoff date, 23 October 2015; hazard ratio, 0.50; 95% CI, 0.40‐0.62; one‐sided *P* < .0001).^13^ The median overall survival (OS) with palbociclib plus fulvestrant vs placebo plus fulvestrant was 34.9 vs 28.0 months (absolute difference, 6.9 months; hazard ratio, 0.81; 95% CI, 0.64‐1.03; *P* = .09).^14^

Because Japanese women were not enrolled in the PALOMA‐1 trial and the PALOMA‐2 results were not yet available, an open‐label, single‐arm, Japanese phase 2 study was initiated to assess the efficacy and safety of palbociclib in combination with letrozole in the treatment of postmenopausal Japanese women with ER+/HER2– ABC.^15^ The probability of PFS at 1 year was 75.0% (90% CI, 61.3‐84.4). However, median PFS was not attained at the primary analysis. After palbociclib was approved by the Ministry of Health, Labour and Welfare in Japan on 27 September 2017, the study continued as a postmarketing clinical study until a sufficient number of PFS events were obtained. In this report, updated efficacy and safety results with a longer follow‐up period are presented from the Japanese phase 2 trial, together with the subsequent therapy and health‐related quality of life (HRQoL) data—the first time these types of data have been reported for Japanese patients from this trial.

## 2 PATIENTS AND METHODS

### 2.1 Study design and patients

This phase 2, single‐arm, open‐label, multicenter study was conducted in Japan. Detailed methods have been previously published.^15^ Patients were postmenopausal women with ER+/HER2– ABC who had received no prior systemic therapy for their advanced disease. Additional key inclusion criteria were age ≥20 years; measureable disease per Response Evaluation Criteria in Solid Tumors (RECIST) v1.1 or bone‐only disease; Eastern Cooperative Oncology Group performance status (ECOG PS) 0‐2; and adequate bone marrow, renal, and liver function. Key exclusion criteria were HER2+ disease; advanced, symptomatic visceral spread; prior (neo)adjuvant treatment with a nonsteroidal AI and disease recurrence during treatment or ≤12 months after completing treatment; and prior treatment with a CDK4/6 inhibitor. The study was approved by the Institutional Review Board of each participating center and conducted according to applicable local laws and regulatory requirements, the International Conference on Harmonisation Good Clinical Practice guidelines, and the Declaration of Helsinki. Written informed consent was obtained from each participant.

### 2.2 Study treatment

Patients received 125mg/d oral palbociclib on days 1‐21 of each 28‐day cycle, followed by 7days without palbociclib treatment. Patients also received 2.5mg/d letrozole administered continuously. Palbociclib dose interruptions/delays were permitted in response to protocol‐specified, treatment‐related adverse events (AEs) that included uncomplicated grade 3 neutropenia, grade 3 neutropenia with fever, grade 4 neutropenia, grade 4 thrombocytopenia, grade ≥3 nonhematologic toxicity, and grade 3 QTc prolongation (QTc ≥501 ms on≥ 2 separate electrocardiograms). If necessary, the first palbociclib dose reduction was from 125 to 100mg/d, and the second dose reduction was from 100 to 75mg/d. After the study transitioned to a postmarketing clinical study, the patients switched from the study drug to commercially available palbociclib.

### 2.3 Outcomes

The primary outcome was 1‐year PFS. Secondary outcomes included PFS, objective response rate (ORR), disease control rate (DCR), duration of objective response (DOR), 1‐year survival, OS, safety, and patient‐reported HRQoL. Safety was recorded during treatment and for 28 days after treatment; severity of AEs was graded using the Common Terminology Criteria for Adverse Events (CTCAE) v4.0 and classified according to the Medical Dictionary for Regulatory Activities (MedDRA, v21.1). Patient‐reported HRQoL was measured using the Functional Assessment of Cancer Therapy‐General (FACT‐G)^16^ and Functional Assessment of Cancer Therapy‐Breast (FACT‐B)^17^ scales, and the Trial Outcome Index (TOI)^18^; questionnaires were completed at baseline, day 1 of cycles 2 and 3, and day 1 of every other following cycle.

### 2.4 Statistical analysis

All efficacy analyses were performed using the full analysis set, which comprised all patients who received >1 dose of palbociclib. Efficacy was also assessed in the baseline disease characteristic subgroups of visceral or nonvisceral metastases; length of disease‐free interval (DFI) since completion of prior treatment (≤12months, >12months, or de novo metastatic disease); prior or no prior endocrine therapy; prior or no prior chemotherapy; age (<65years or ≥65 years); and Ki67 staining (≤20% or >20%). The 1‐year PFS probability and 90% CI were estimated using the Kaplan‐Meier method. Median PFS, OS, and associated 95% CIs were also estimated using the Kaplan‐Meier method. ORR was defined as the percentage of patients who had either complete response (CR) or partial response (PR) based on RECIST criteria. DCR was the percentage of patients with CR, PR, or stable disease lasting ≥24 weeks. DOR was analyzed in patients in the full analysis set with OR and defined as the time from first OR to first objective disease progression or death. Treatment duration of subsequent therapy was estimated using the Kaplan‐Meier method. Change from baseline in HRQoL scale scores was summarized by descriptive statistics (mean±SD). As a post hoc analysis, an examination of the time to definitive deterioration (TTD) in FACT‐B total score was conducted using the Kaplan‐Meier method. A meaningful change in QOL is defined as a change in baseline score equal to or greater than the established minimally important differences: for FACT‐B, this is 7‐8 points.^19^ In this study, TTD was defined as the duration between baseline and first occurrence of a decrease of ≥7 points in FACT‐B score with no subsequent observation of a >7‐point decrease, which is the same definition as that used in PALOMA‐2.^20^

## 3 RESULTS

### 3.1 Patient population

A total of 42 patients were enrolled in the study. Patient demographics and baseline disease characteristics have been previously published^15^; brief patient demographics and baseline disease characteristics are presented in Table 1. Median patient age was 62.5 years. Approximately half of the patients had visceral metastases (47.6%). Eight patients (19.0%) had a DFI ≤12 months.

**Table 1:** Patient demographics and baseline disease characteristics

Characteristic	Palbociclib + Letrozole N = 42
Age, median (range), y	62.5 (43‐84)
Weight, median (range), kg	50.4 (38.6‐74.5)
ECOG PS, n (%)	
0	39 (92.9)
1	3 (7.1)
Disease site, n (%)	
Visceral	20 (47.6)
Nonvisceral	22 (52.4)
Bone only	6 (14.3)
DFI, n (%)	
≤12 mo	8 (19.0)
>12 mo	20 (47.6)
De novo metastatic	14 (33.3)
Prior (neo)adjuvant therapies, n (%)	
Hormone therapy	27 (64.3)
Chemotherapy	20 (47.6)
Ki67‐positive expression, n (%)	
≤20%	19 (45.2)
>20%	23 (54.8)

Abbreviations: DFI, disease‐free interval; ECOG PS, Eastern Cooperative Oncology Group performance status.

### 3.2 Drug exposure

Study drug exposure is summarized in Table2. At a data cutoff date of 25 October 2018, 13 patients (31.0%) were being treated with commercially available palbociclib plus letrozole or letrozole alone. The median duration of study treatment was 33.0months (range, 1.8‐49.2). Median relative palbociclib dose intensity was 70.7% (range, 38.0‐99.6). In total, 29 patients (69.0%) required ≥1 palbociclib dose reduction due to an AE: 16 were reduced to 100mg/d, and 12 were reduced to 75mg/d. In addition, 1 patient reduced to 100mg/d and mistakenly took 25mg×2 capsules (=50mg) instead of 25mg×4 capsules (=100mg) for 1day. Palbociclib dose interruption was experienced by 39 patients (92.9%). The mean (SD) number of interruptions per patient was 2.7 (2.0), and the mean (SD) duration of the dose interruptions was 4.6 (2.3) days. Thirty‐two patients (76.2%) required dose interruption due to an AE. Palbociclib cycle delay occurred in 40 patients (95.2%); the mean (SD) number of cycle delays per patient was 13.6 (11.9), and the mean (SD) duration of cycle delay was 9.3 (6.6) days. Thirty‐nine patients (92.9%) required cycle delay due to an AE.

**Table 2:** Exposure to study drug

	Palbociclib + Letrozole N = 42
Duration of treatment,^a^ median (range), months	33.0 (1.8‐49.2)
Palbociclib (N = 42)	
Average daily dose, median (range), mg	100.6 (75‐125)
Dose reductions,^b^ n (%)	29 (69.0)
Reduction to 100 mg	16 (38.1)
Reduction to 75 mg	12 (28.6)
Reduction to 50 mg	1 (2.4)^f^
Time to first dose reduction,^c^ median (range), days	66 (29‐1067)
Dose interruption,^d^ n (%)	39 (92.9)
Cycle delay,^e^ n (%)	40 (95.2)
Relative dose intensity, median (range), %	70.7 (38.0‐99.6)
Letrozole (N = 42)	
Dose interruption,^d^ n (%)	35 (83.3)
Cycle delay,^e^ n (%)	10 (23.8)
Relative dose intensity, median (range), %	99.7 (69.6‐100.0)

^a^ Total number of days from first to and including last day of each study treatment.

^b^ Includes any dose reduction from the initial prescribed dose not including dose interruptions.

^c^ Timed from (start of first occurrence minus first dose date of cycle 1) + 1.

^d^ Interruptions include missed dose based on the case report form and dose administered = 0 mg.

^e^ Cycle delay is defined as any delay of the cycle start date.

^f^ One patient mistakenly took 25 mg × 2 capsules (=50 mg) instead of 25 mg × 4 capsules (=100 mg) for 1 d.

### 3.3 Efficacy

**Figure 1:**
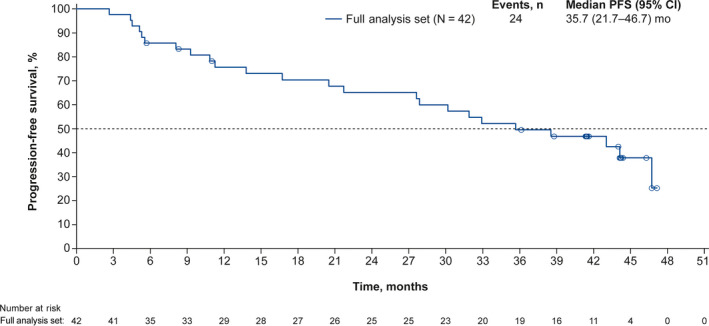
Kaplan‐Meier estimated PFS probability in the full analysis set. CI, confidence interval; PFS, progression‐free survival

**Figure 2:**
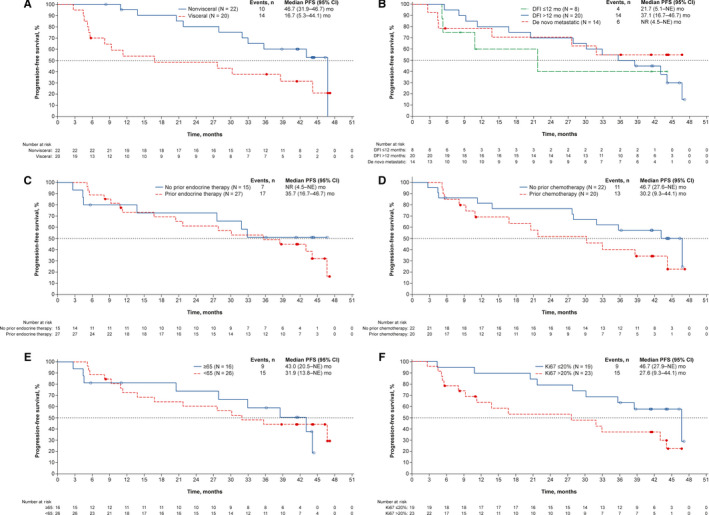
Kaplan‐Meier estimated PFS probability by patient subgroup. A, Visceral and nonvisceral metastasis subgroups. B, DFI since prior (neo)adjuvant therapy subgroups. C, Prior and no prior endocrine therapy subgroups. D, Prior and no prior chemotherapy subgroups. E, Age <65 y and ≥65 y subgroups. F, Ki67 expression subgroups. CI, confidence interval; DFI, disease‐free interval; NE, not estimable; NR, not reached; PFS, progression‐free survival

**Figure 3:**
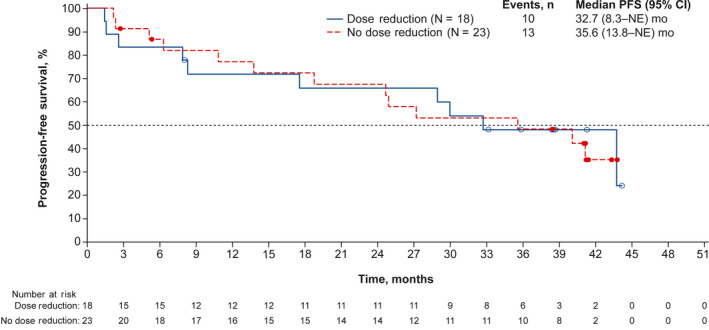
Kaplan‐Meier estimated PFS probability for patients with and without dose reduction during the first 90 d. Patients with PFS time within 90 d were excluded. CI, confidence interval; NE, not estimable; PFS, progression‐free survival

At the data cutoff date, the 1‐year probability of PFS was 75.6% (90% CI, 62.4‐84.7). The Kaplan‐Meier estimated median PFS was 35.7months (95% CI, 21.7‐46.7; Figure1). PFS was also assessed in subgroups based on baseline characteristics (Figure2). Median PFS was >40 months for patients with nonvisceral metastatic disease, for patients without prior chemotherapy, for patients with age ≥65 years, and for patients with Ki67 ≤20%. Median PFS was not reached for patients with de novo metastatic disease and for patients without prior endocrine therapy. A landmark analysis was performed to assess PFS in patients who did and did not require dose reduction within the first 90days (Figure3); PFS was similar in both subgroups.

**Figure 4:**
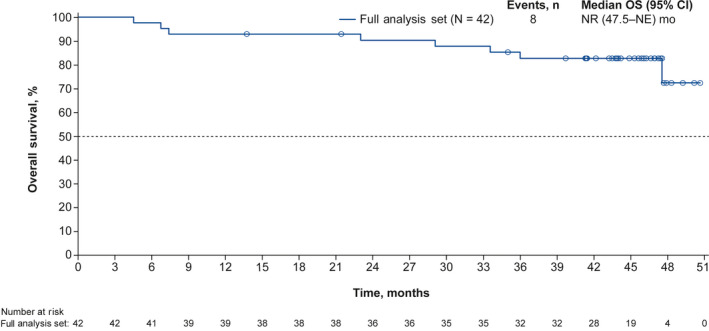
Kaplan‐Meier estimated OS probability in the full analysis set. CI, confidence interval; NE, not estimable; NR, not reached; OS, overall survival

In the full analysis set, 20 patients had OR, and the median DOR was 41.4 months (95% CI, 19.0–not estimable [NE]). In the full analysis set, the ORR was 47.6% (95% CI, 32.0‐63.6) and the DCR was 85.7% (95% CI, 71.5‐94.6). In the response evaluable set (n = 36), the ORR and DCR were 55.6% (95% CI, 38.1‐72.1) and 83.3% (95% CI, 67.2‐93.6), respectively. OS data remain immature; median OS was not reached at the date of this data cutoff (Figure 4). Survival probability was 92.9% (95% CI, 79.5‐97.6) at 1 year, 90.3% (95% CI, 76.3‐96.3) at 2 years, and 82.7% (95% CI, 67.1‐91.4) at 3 years.

### 3.4 Subsequent therapy

**Figure 5:**
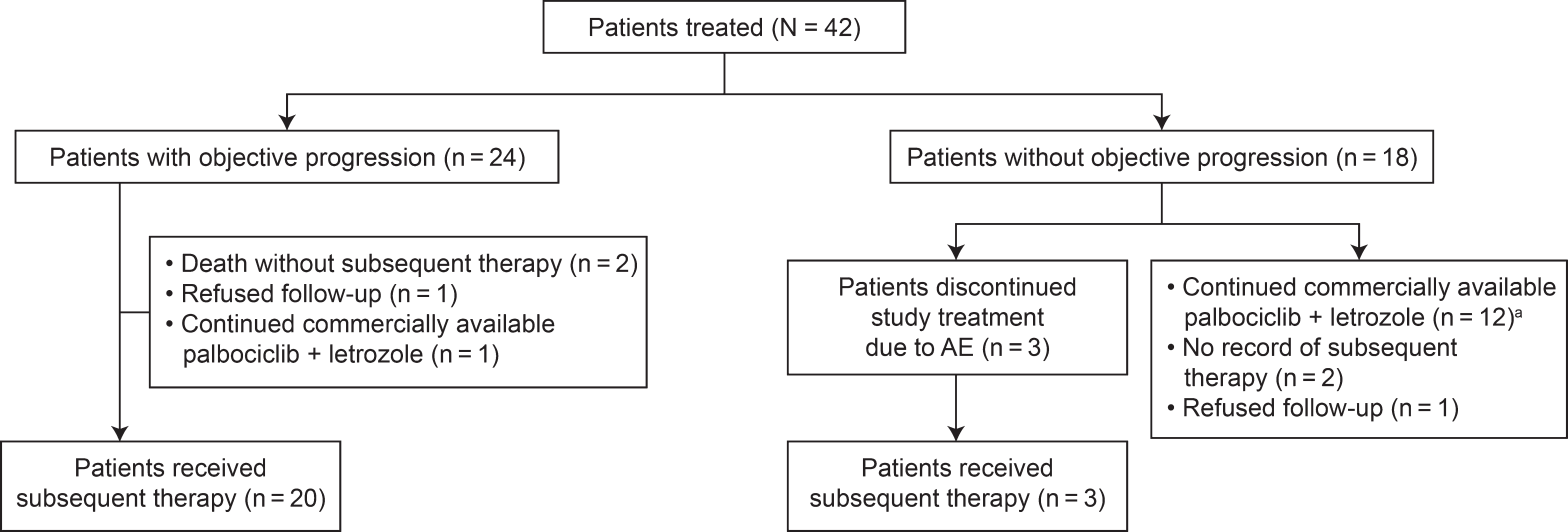
CONSORT diagram of subsequent therapy. ^a^One patient continued commercially available letrozole only. AE, adverse event

Of the 42 patients enrolled in the study, 24 patients had PFS events at the data cutoff date. Four of 24 patients did not have a record of subsequent therapy. Three patients permanently discontinued the study treatment due to AEs and then received subsequent therapy. In total, 23 patients (54.8%) received subsequent systemic anticancer therapies (Figure 5).

Among these 23 patients, 10 received 1 subsequent regimen, 2 received 2 subsequent regimens, 3 received 3 subsequent regimens, and 8 received >3 subsequent regimens at the data cutoff date. Of the 42 patients, 13 patients were being treated with commercially available palbociclib plus letrozole or letrozole alone, and these patients were not included in the analyses of subsequent therapy assessment.

The most common first subsequent therapy was endocrine therapy, followed by chemotherapy (20 patients [87.0%] and 3 patients [13.0%], respectively; Table 3). Fulvestrant alone or fulvestrant‐containing regimens were used in 14 patients (60.9%). Exemestane alone or exemestane‐containing regimens were used in 3 patients (13.0%); everolimus plus exemestane was used in 1 of these 3 patients. Paclitaxel alone or paclitaxel‐containing regimens were used in 3 patients (paclitaxel alone in 1 patient, and bevacizumab plus paclitaxel in 2 patients) (Table S1).

**Table 3:** Summary of subsequent anticancer treatment regimens

Systemic Anticancer Therapy, n (%)	Palbociclib + Letrozole N = 23^a^
First subsequent therapy	23 (100.0)
Endocrine therapy	20 (87.0)
Chemotherapy	3 (13.0)
VEGF inhibitor	2 (8.7)
CDK4/6 inhibitor	2 (8.7)
mTOR inhibitor	1 (4.3)
Other	2 (8.7)
Second subsequent therapy	13 (56.5)
Endocrine therapy	7 (30.4)
Chemotherapy	5 (21.7)
VEGF inhibitor	0
CDK4/6 inhibitor	1 (4.3)
mTOR inhibitor	0
Other	2 (8.7)
Third or greater subsequent therapy	11 (47.8)
Endocrine therapy	9 (39.1)
Chemotherapy	10 (43.5)
VEGF inhibitor	4 (17.4)
CDK4/6 inhibitor	1 (4.3)
mTOR inhibitor	4 (17.4)
Other	2 (8.7)

Abbreviations: CDK4/6, cyclin‐dependent kinase 4/6; mTOR, mammalian target of rapamycin; VEGF, vascular endothelial growth factor.

^a^ Total number of patients who received subsequent therapy.

**Figure 6:**
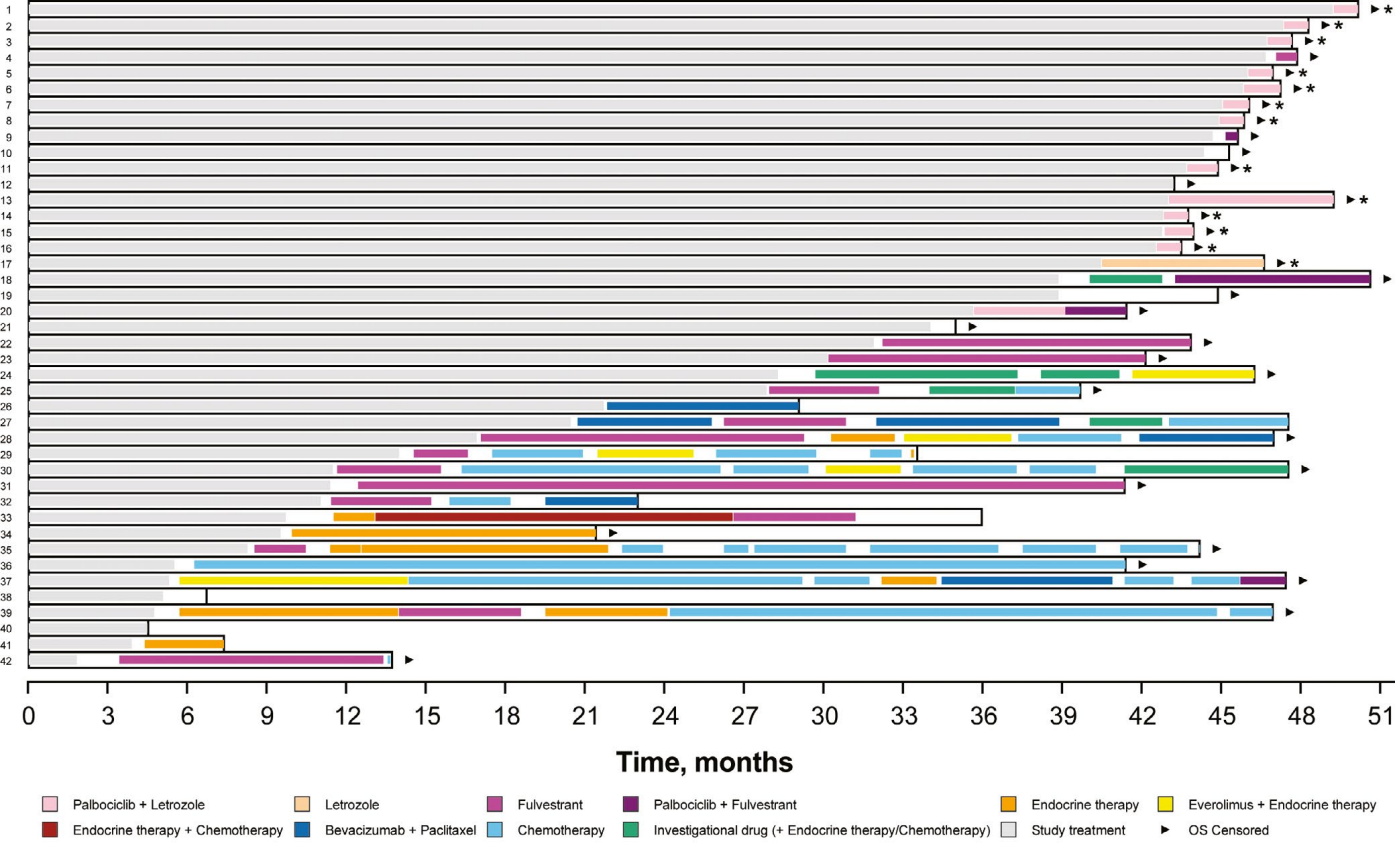
Time course of subsequent therapies in each patient. *Patients who were being treated with commercially available palbociclib plus letrozole or letrozole alone at the data cutoff date. OS, overall survival

Median duration of first, second, and third or greater subsequent therapy was 8.3 (95% CI, 3.9‐12.2), 3.5 (95% CI, 2.3‐9.8), and 3.8 (95% CI, 2.8‐4.6) months, respectively. Five patients received an everolimus‐containing regimen (4 patients and 1 patient received everolimus plus exemestane and everolimus plus toremifene, respectively) as any line of subsequent therapy. Median duration of subsequent everolimus‐containing treatment was 4.1 months (95% CI, 2.1‐8.4). The median time to first use of subsequent chemotherapy was not yet reached (95% CI, 24.2 months—NE) at the data cutoff date (Figure S1). The time course of subsequent therapies in each patient is shown in Figure 6.

### 3.5 Safety

The most commonly reported AEs were similar to those previously described (Table 4), and no new safety signals were observed with longer follow‐up. Neutropenia remained the most frequent AE; however, treatment‐related febrile neutropenia (grade 3) was only observed in 1 patient. Two patients received granulocyte‐colony stimulating factor (G‐CSF), 1 due to grade 4 neutropenia and the other due to grade 3 febrile neutropenia. After recovery, both patients resumed palbociclib at a lower dose level. Two treatment‐related serious AEs (SAEs) were previously reported in this study: 1 report of grade 3 febrile neutropenia and 1 report of grade 5 subarachnoid hemorrhage. For this patient, the last doses of palbociclib and letrozole were given 12 and 5 days, respectively, before death.^15^ No new treatment‐related SAEs have been reported since the primary analysis. Seven of the 42 patients (16.7%) discontinued palbociclib treatment due to treatment‐related AEs (neutropenia in 4 patients; and malaise, alanine aminotransferase/aspartate aminotransferase increased and subarachnoid hemorrhage in 1 patient each).

**Table 4:** Palbociclib treatment‐related AEs occurring in ≥10% of patients

AE, n (%)	Palbociclib + Letrozole N = 42	
	Any Grade	Grade 3/4
Any AE	42 (100.0)	39 (92.9)
Hematologic AE		
Neutropenia^a^	42 (100.0)	39 (92.9)
Leukopenia^b^	35 (83.3)	25 (59.5)
Thrombocytopenia^c^	11 (26.2)	0
Anemia	9 (21.4)	2 (4.8)
Nonhematologic AE		
Stomatitis^d^	32 (76.2)	0
Infection^e^	11 (26.2)	0
Rash^f^	10 (23.8)	0
Constipation	10 (23.8)	0
ALT increased	10 (23.8)	4 (9.5)
AST increased	10 (23.8)	1 (2.4)
Alopecia	8 (19.0)	0
Malaise	7 (16.7)	0
Headache	6 (14.3)	0
Diarrhea	5 (11.9)	0
Epistaxis	5 (11.9)	0
Nausea	5 (11.9)	0

Abbreviations: AE, adverse event; ALT, alanine aminotransferase; AST, aspartate aminotransferase; MedDRA, Medical Dictionary for Regulatory Activities; PT, preferred term.

^a^ Neutropenia was categorized according to the MedDRA PTs neutropenia and neutrophil count decreased.

^b^ Leukopenia was categorized according to the MedDRA PTs leukopenia and white blood cell count decreased.

^c^ Thrombocytopenia was categorized according to the MedDRA PTs thrombocytopenia and platelet count decreased.

^d^ Stomatitis was categorized according to the MedDRA PTs cheilitis, glossitis, mucosal inflammation, oropharyngeal pain, and stomatitis.

^e^ Infections were categorized according to the MedDRA PTs angular cheilitis, cellulitis, conjunctivitis, cystitis, genital candidiasis, gingivitis, laryngitis, lip infection, nasopharyngitis, oral herpes, otitis media, pharyngitis, and upper respiratory tract infection.

^f^ Rash was categorized according to the MedDRA PTs rash, rash maculopapular, dermatitis, and dermatitis acneiform.

### 3.6 HRQoL

**Figure 7:**
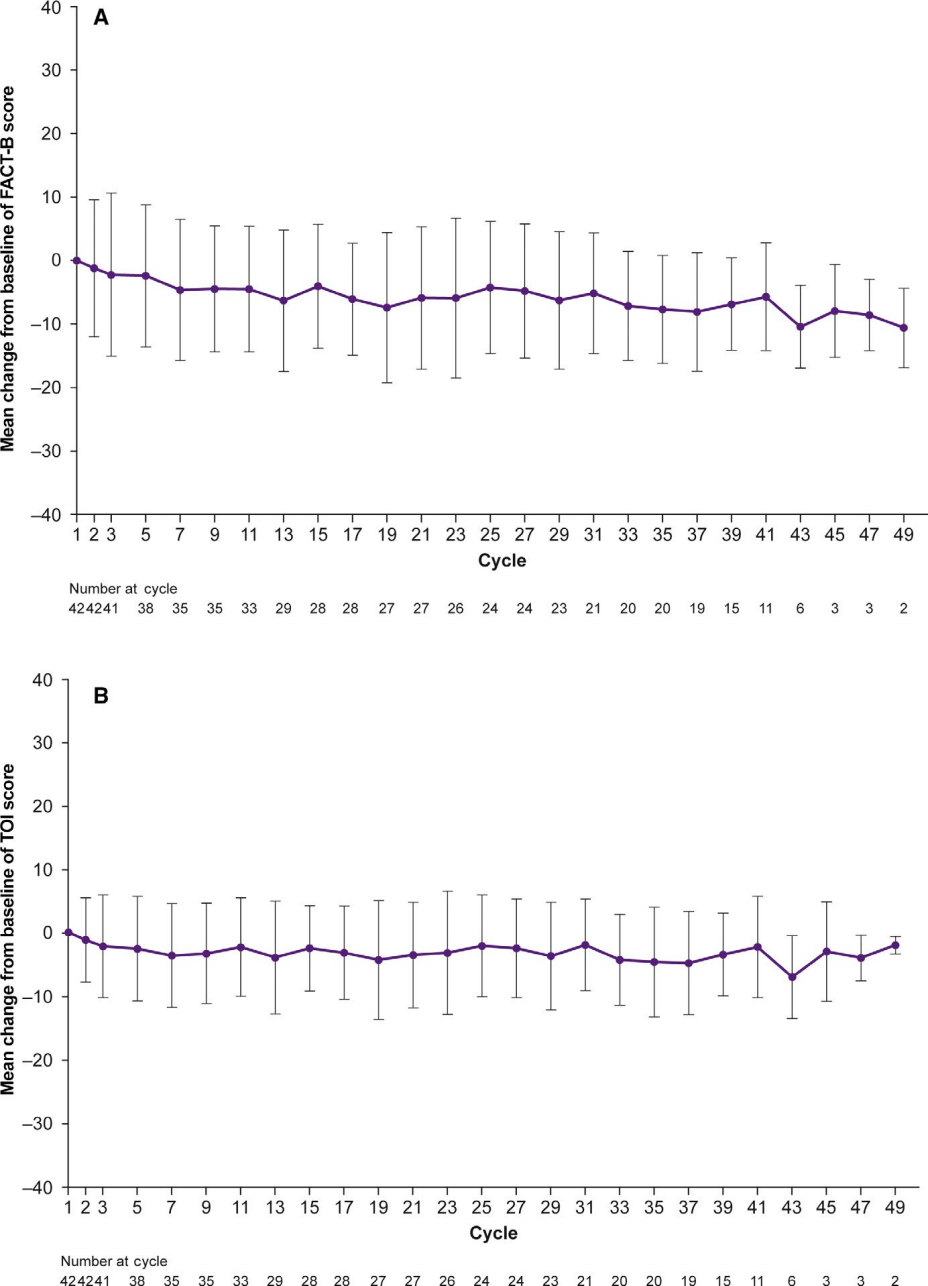
Change from baseline in patient‐reported HRQoL scale scores. A, FACT‐B total score. B, TOI total score. Bars indicate standard deviations. FACT‐B, Functional Assessment of Cancer Therapy‐Breast; HRQoL, health‐related quality of life; TOI, Trial Outcome Index

**Figure 8:**
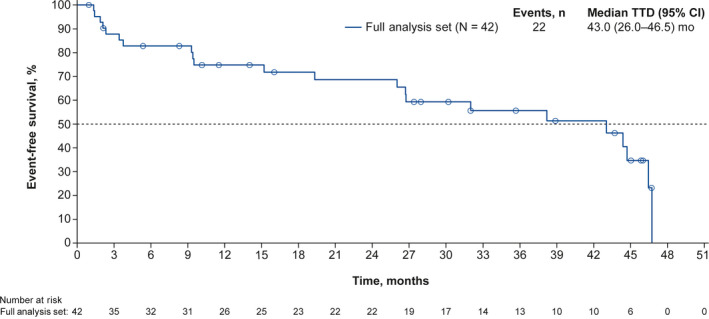
Kaplan‐Meier estimated TTD probability in FACT‐B score. CI, confidence interval; FACT‐B, Functional Assessment of Cancer Therapy‐Breast; TTD, time to definitive deterioration

Overall changes from baseline to end of study treatment in FACT‐B and TOI total scores remained consistent (Figure 7), indicating that HRQoL was maintained with palbociclib plus letrozole. Changes from baseline to the end of study treatment in FACT‐G total scores and FACT‐B subscale scores were also consistent over the course of the study (Figure S2). The Kaplan‐Meier estimated median TTD in FACT‐B score was 43.0 months (95% CI, 26.0‐46.5; Figure 8).

## 4 DISCUSSION

With extended follow‐up, palbociclib plus letrozole remains an effective and well‐tolerated therapy in postmenopausal Japanese women with ER+/HER2– ABC. In general, the data reported here are consistent with those of the PALOMA‐2 extended follow‐up results; with a median duration of follow‐up of 37.6 months (data cutoff date, 31 May 2017), median PFS in the palbociclib arm of PALOMA‐2 was 27.6 months (95% CI, 22.4‐30.3).^10^ Patient baseline disease characteristics were similar between the 2 studies, although the proportion of patients with ECOG PS of 0 was higher in the Japanese phase 2 study than in PALOMA‐2 (92.9% and 57.9%, respectively).^10^ Overall, palbociclib plus letrozole appears to be as effective in Japanese patients as in the overall population of PALOMA‐2.

Palbociclib plus letrozole remained well tolerated in Japanese patients. No new safety signals were observed in Japanese patients with longer follow‐up. Hematologic AEs were the most frequently reported AEs with extended follow‐up in both this Japanese phase 2 study and in PALOMA‐2.^10^ The rate of all‐grade neutropenia was higher in the Japanese phase 2 study and in the Japanese subgroup analysis of PALOMA‐2 than in the overall population in PALOMA‐2.^9^, ^10^ In this Japanese phase 2 study, neutropenia was reported in 100% of patients receiving palbociclib. However, Japanese patients in the phase 2 study reported similar rates of treatment‐related SAEs (4.8%) as did patients receiving palbociclib in PALOMA‐2 (6.5%).^10^ In both the Japanese study and in PALOMA‐2, febrile neutropenia was rare, reported in 2.4% and 2.0% of patients, respectively. Palbociclib‐related toxicities were generally managed with dose modification in Japanese patients. It was reported that dose reduction of palbociclib did not appear to impact the efficacy at the primary analysis^15^; however, the data were immature and we could not fully evaluate the impact of dose reduction on efficacy. In this report, the landmark analysis with extended follow‐up showed that PFS was similar in Japanese patients regardless of whether they required a palbociclib dose reduction within the first 90 days of treatment. Importantly, palbociclib tolerability appears to be similar in Japanese patients and in patients of other ethnicities, and dose modifications to manage palbociclib toxicities are unlikely to affect the efficacy in Japanese patients.

Japanese patients with nonvisceral metastases demonstrated greater benefit with palbociclib plus letrozole than did patients with visceral metastases; median PFS was approximately 2.5years longer in patients with nonvisceral metastases (46.7 and 16.7months). A similar PFS difference between patients with visceral and nonvisceral metastases was also observed in other clinical trials. The proportion of patients with nonvisceral metastases was comparable in the Japanese phase 2 study and PALOMA‐2 (52.4% and 51.8%, respectively). In PALOMA‐2, median PFS was >1year longer in patients with nonvisceral metastases compared with patients with visceral metastases (35.9 and 19.3months, respectively).^10^ Similarly in PALOMA‐3, median PFS was approximately 3months longer in patients with nonvisceral vs visceral disease (11.2 and 8.0months, respectively).^21^ Median PFS was also longer for Japanese patients with a DFI >12 months than for patients with a shorter DFI; the difference between the 2 groups was >1 year (37.1 vs 21.7months). Median PFS was not reached for the 14 patients with de novo metastatic disease. Kaplan‐Meier plots of PFS were similar between Japanese patients with or without prior endocrine therapy. In contrast, median PFS was longer in Japanese patients who did not have prior chemotherapy compared to those with prior chemotherapy. Median PFS was also longer in Japanese patients aged ≥65 years vs those <65 years of age. Of note, in PALOMA‐2, median PFS was prolonged with palbociclib compared with placebo in all patient subgroups examined, suggesting that all patient subpopulations derive benefit from the addition of palbociclib to letrozole, regardless of their baseline characteristics.^10^ In Japanese patients, median PFS was longer for the patients with Ki67 expression ≤20% vs >20% (46.7 and 27.6months, respectively). In PALOMA‐2, Ki‐67 index values at a 15% or 20% cutoff did not reveal a patient group with a better or worse PFS with palbociclib plus letrozole compared with placebo plus letrozole.^22^ These subgroup data support the efficacy of palbociclib plus letrozole across patient subgroups.

Most Japanese patients (87%) received endocrine therapy as their first subsequent therapy after disease progression on palbociclib, and approximately 30% received endocrine therapy as their second subsequent therapy. Only 3 patients (13%) received chemotherapy as their first subsequent therapy. Single‐agent fulvestrant was the most common subsequent treatment. In PALOMA‐2, 60.8% and 36.6% of patients received endocrine therapy and chemotherapy, respectively, as the first subsequent treatment after discontinuation of palbociclib,^10^ suggesting that patients enrolled in PALOMA‐2 may have had more aggressive disease than did the patients in the Japanese study. However, PALOMA‐2 was a large, multinational trial, and differences in approved drug and insurance/reimbursement systems among countries could influence the selection of subsequent treatments after the discontinuation of palbociclib. Importantly, the data from the Japanese phase 2 study suggest that endocrine therapy remains effective after palbociclib combination therapy in the majority of Japanese patients. Subsequent endocrine therapy is advantageous because it delays the use of chemotherapy treatment, which is associated with increased toxicity and reduced quality of life.

Generally, no clinically meaningful deterioration in FACT‐G, FACT‐B, and TOI total scores and FACT‐B subscale scores was observed in Japanese patients in this study. This finding is similar to that observed in the overall PALOMA‐2 study population with extended follow‐up, in which the FACT‐B total score was maintained over time with palbociclib plus letrozole, and changes from baseline did not significantly differ between the palbociclib and placebo arms.^10^ In addition, median TTD was 43.0 months in this study. This is longer than the findings from PALOMA‐2 in which the same definition of TTD was used for the analysis (24.9 months in the placebo plus letrozole arm and 26.3 months in the palbociclib plus letrozole arm).^20^ These results suggest that palbociclib treatment does not negatively impact QoL in Japanese patients.

This study is subject to certain limitations, including its single‐arm, open‐label design and small sample size, and therefore may be subject to biases. However, with extended follow‐up, treatment of Japanese patients with ER+/HER2– ABC with palbociclib in combination with letrozole yielded a median PFS of almost 3 years. Palbociclib plus letrozole was confirmed as safe and effective, without clinically meaningful deterioration in patient‐reported HRQoL, in postmenopausal Japanese patients with ER+/HER2– ABC. Furthermore, as the majority of patients received hormonal therapy as subsequent therapy after disease progression with palbociclib plus letrozole in this study, it will provide the opportunity for future analysis of the efficacy of subsequent therapies.

## AUTHOR CONTRIBUTIONS

M. Takahashi, H. Iwata, S. Hashigaki, Y. Muramatsu, Y. Umeyama, and M. Toi made substantial contributions to the study conception and design. M. Takahashi, N. Masuda, R. Nishimura, K. Inoue, S. Ohno, H. Iwata, and M. Toi participated in data acquisition. S. Hashigaki performed data analysis. All authors made substantial contributions to the interpretation of data. All authors were involved in drafting the manuscript or revising it critically for important intellectual content. All authors read and approved the final version of the manuscript to be published; have participated sufficiently in the work to take public responsibility for appropriate portions of the content; and agree to be accountable for all aspects of the work in ensuring that questions related to the accuracy or integrity of any part of the work are appropriately investigated and resolved.

## CONFLICTS OF INTEREST

Authors SH and YU are employees of Pfizer R&D Japan. YU also owns stock in Pfizer Inc. YM is an employee of Pfizer Japan Inc. HI received honoraria from Chugai, Daiichi‐Sankyo, AstraZeneca, and Pfizer; manuscript fees from Eisai; and research funding from MSD, Chugai, Daiichi‐Sankyo, and Boehringer Ingelheim. KI’s institution received research funding from Parexel, Puma Biotechnology, Pfizer, Novartis, MSD, GSK, Chugai, and Daiichi‐Sankyo. NM received honoraria from Chugai, Pfizer, Eli Lilly, Eisai, Takeda, and AstraZeneca; is a board member of the Japan Breast Cancer Research Group Association; and his institution received research funding from Chugai, AstraZeneca, Kyowa‐Kirin, MSD, Novartis, Pfizer, Eli Lilly, Eisai, and Daiichi‐Sankyo. SO received honoraria from Chugai, AstraZeneca, Eisai, Pfizer, Taiho, Kyowa‐Kirin, Nippon Kayaku, and Novartis, and research funding from Taiho and Eisai. RN received honoraria from Pfizer, Novartis, and Chugai. MTa received honoraria from Pfizer, AstraZeneca, Eli Lilly, and Eisai; and research funding from Taiho, Kyowa‐Kirin, and Eisai. MTo received honoraria from AstraZeneca and Eli Lilly; research funding from Taiho, Kyowa‐Kirin, Shimadzu, AFF Technology, Bizcom Japan, C&C Research Laboratories, Astellas, AstraZeneca, and Chugai; scholarship endowments from Chugai, Eisai, and Pfizer; and is a member of the board (no salary) of the Japan Breast Cancer Research Group association, Kyoto Breast Cancer Research Network, and Organisation for Oncology and Translational Research.

## Supporting information

Supplementary Material

## Data Availability

Upon request, and subject to certain criteria, conditions, and exceptions (see https://www.pfizer.com/science/clinical‐trials/trial‐data‐and‐results for more information), Pfizer will provide access to individual deidentified participant data from Pfizer‐sponsored global interventional clinical studies conducted for medicines, vaccines, and medical devices (1) for indications that have been approved in the US and/or EU or (2) in programs that have been terminated (ie, development for all indications has been discontinued). Pfizer will also consider requests for the protocol, data dictionary, and statistical analysis plan. Data may be requested from Pfizer trials 24 months after study completion. The deidentified participant data will be made available to researchers whose proposals meet the research criteria and other conditions, and for which an exception does not apply, via a secure portal. To gain access, data requestors must enter into a data access agreement with Pfizer.
