# Supplementary material for: Palbociclib‐letrozole as first‐line treatment for advanced breast cancer: Updated results from a Japanese phase 2 study
Source: Cancer Med. 2020 May 18;9(14):4929–40. doi: 10.1002/cam4.3091 (PMC7367628; doi:10.1002/cam4.3091)
Supplement: Supplementary file 1 — Supplementary Material [file CAM4-9-4929-s001.pdf]

Table S1. Summary of Subsequent Anticancer Treatment Regimens by Combination

Systemic Anticancer Therapy, n (%)	Palbociclib + Letrozole n=23[a]
First subsequent therapy	23 (100.0)
Bevacizumab + paclitaxel	2 (8.7)
Everolimus + exemestane	1 (4.3)
Exemestane	1 (4.3)
Exemestane + investigational drug	1 (4.3)
Fulvestrant	11 (47.8)
Fulvestrant + investigational drug	1 (4.3)
Fulvestrant + palbociclib	2 (8.7)
Medroxyprogesterone	1 (4.3)
Paclitaxel	1 (4.3)
Tamoxifen	2 (8.7)
Second subsequent therapy	13 (56.5)
Capecitabine	2 (8.7)
Exemestane + investigational drug	1 (4.3)
Fulvestrant	2 (8.7)
Fulvestrant + palbociclib	1 (4.3)
Investigational drug	1 (4.3)
Medroxyprogesterone	1 (4.3)
Tamoxifen	2 (8.7)
TS-1	3 (13.0)
Third or greater subsequent therapy	11 (47.8)
Bevacizumab + paclitaxel	4 (17.4)
Capecitabine	2 (8.7)
Capecitabine + cyclophosphamide	1 (4.3)
Cyclophosphamide	1 (4.3)
Cyclophosphamide + doxorubicin	1 (4.3)
Cyclophosphamide + epirubicin	3 (13.0)
Docetaxel	1 (4.3)
Eribulin	3 (13.0)
Everolimus + exemestane	3 (13.0)
Everolimus + toremifene	1 (4.3)
Exemestane + investigational drug	1 (4.3)
Fulvestrant	1 (4.3)
Fulvestrant + palbociclib	1 (4.3)
Gemcitabine	1 (4.3)
Gemcitabine + vinorelbine	1 (4.3)
Investigational drug	1 (4.3)
Medroxyprogesterone	4 (17.4)
Paclitaxel	5 (21.7)
Tegafur	1 (4.3)
Toremifene	1 (4.3)
TS-1	2 (8.7)
Vinorelbine	1 (4.3)

[a]Total number of patients who received subsequent therapy.

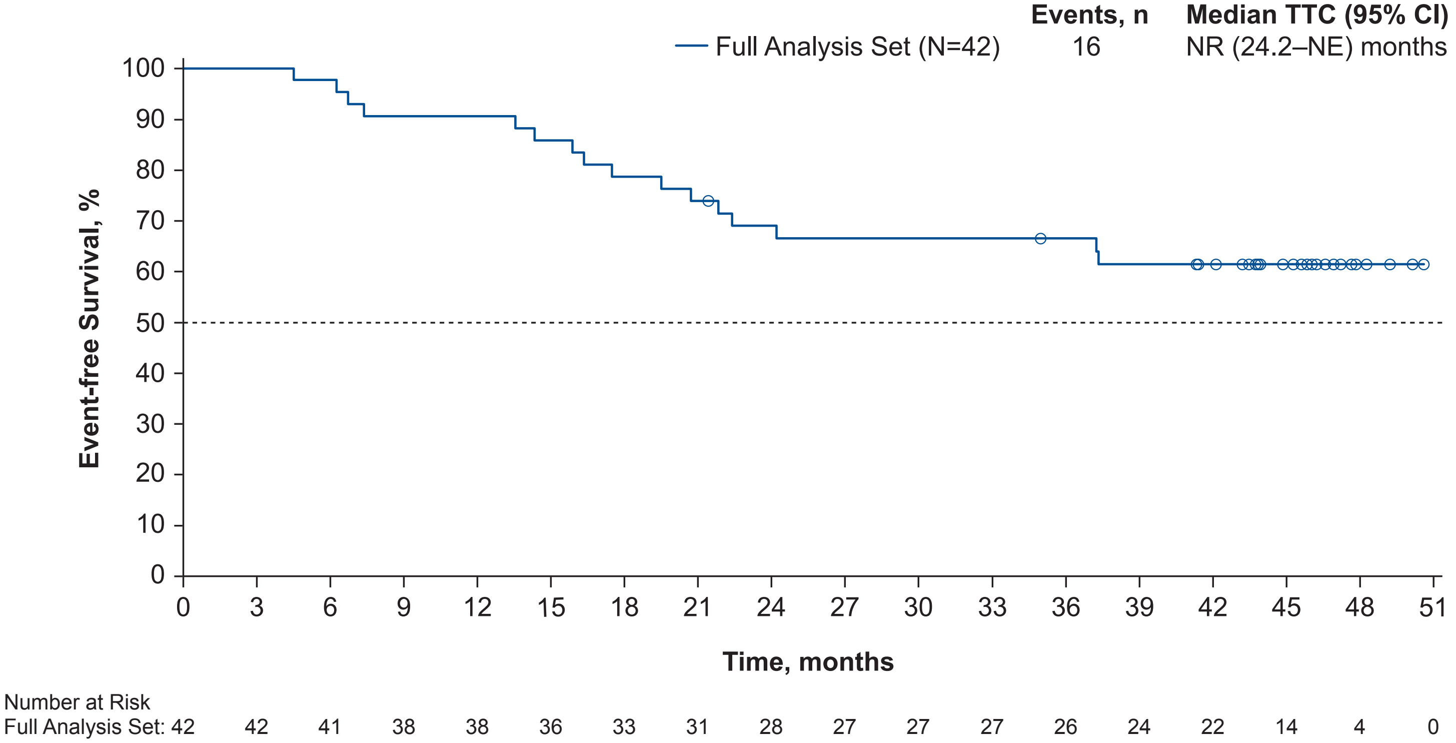

Figure S1. Kaplan-Meier Estimated Time to First Use of Subsequent Chemotherapy.
CI=confidence interval; NE=not estimable; NR=not reached; TTC=time to first subsequent chemotherapy.

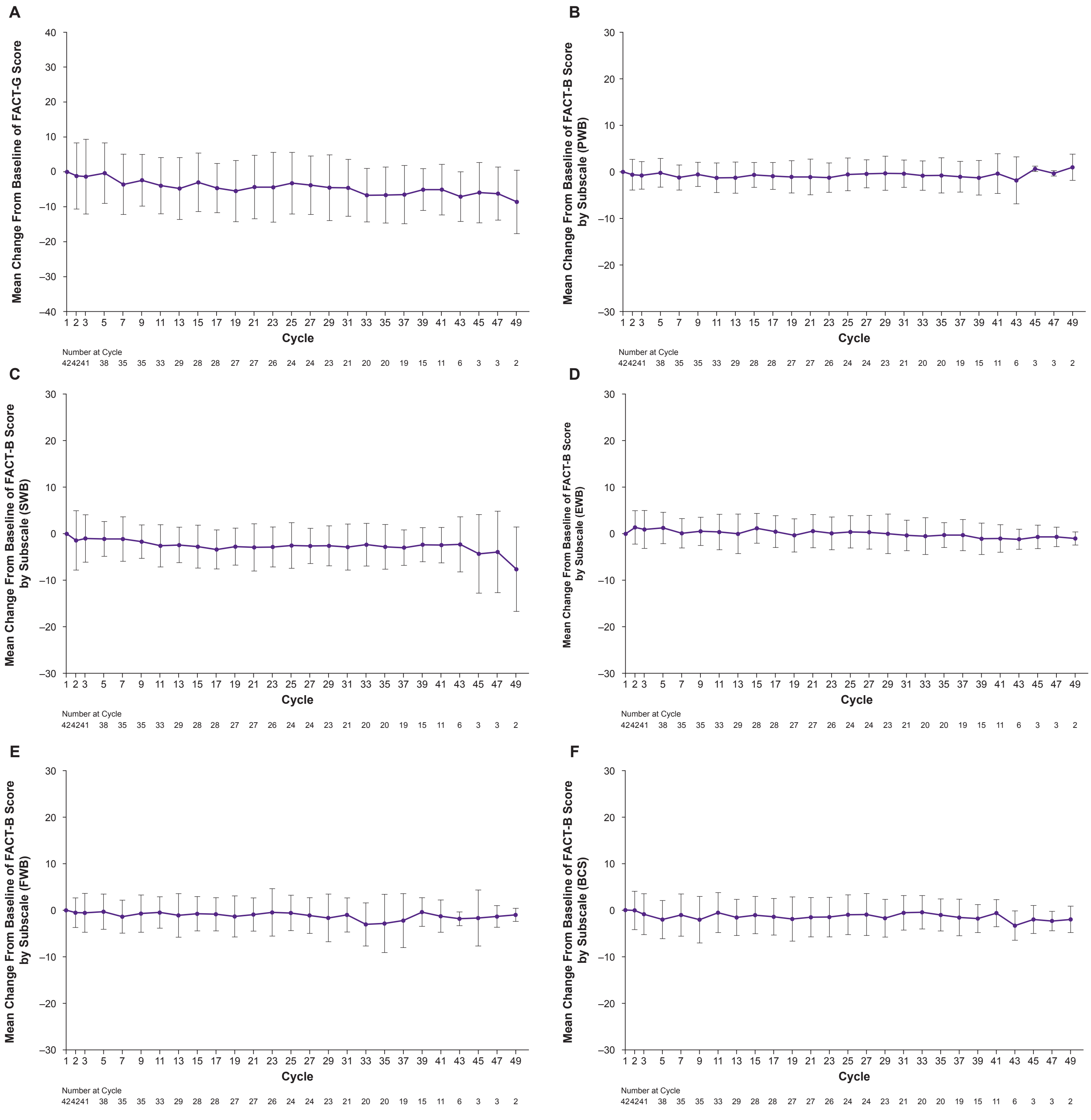

Figure S2. Change From Baseline in Patient-Reported HRQoL Scale Scores.

(A) FACT-G total score. (B) FACT-B PWB subscale score. (C) FACT-B SWB subscale score. (D) FACT-B EWB subscale score. (E) FACT-B FWB subscale score. (F) FACT-B BCS subscale score.

Bars indicate standard deviations. BCS=breast cancer subscale; EWB=emotional well-being; FACT-B=Functional Assessment of Cancer Therapy – Breast; FACT-G=Functional Assessment of Cancer Therapy – General; FWB=functional well-being; HRQoL=health-related quality of life; PWB=physical well-being; SWB=social/family well-being.
